# Effect of mobile-based cognitive behavior therapy (CBT) on lowering of blood lipid levels in atherosclerotic cardiovascular disease (ASCVD) patients: study protocol for a multicenter, prospective, randomized controlled trial

**DOI:** 10.1186/s13063-022-06459-7

**Published:** 2022-06-30

**Authors:** Xu-Lin Hong, Yi Luan, Hong-Ying Liu, Wen-Bin Zhang

**Affiliations:** Sir Run Run Shaw Hospital, Zhejiang University, Hangzhou, Zhejiang China

**Keywords:** Atherosclerotic cardiovascular disease, Low-density lipoprotein cholesterol, Mobile-based cognitive behavior therapy, Compliance

## Abstract

**Background:**

Atherosclerotic cardiovascular disease (ASCVD) remains a major source of mortality in China. Convincing evidence has demonstrated that the reduction of low-density lipoprotein cholesterol (LDL-C) is correlated with lowering ASCVD risk. The efficacy of lifestyle management in lipid levels reduction has been confirmed in numerous studies. However, considering that low compliance to lifestyle management has limited the benefits of lowering lipid levels, cognitive behavior therapy (CBT) is proposed as a solution to improve clinical outcomes. The objective of this trial is to compare the LDL-C outcome in ASCVD patients receiving mobile device-based CBT to a control group, with both groups under standard pharmacological treatments.

**Methods:**

This trial is designed as a multicenter, prospective randomized controlled trial with a 6-month follow-up. Mean LDL-C level and the percentage of different LDL-C levels, coefficient of variation of LDL, General Self-Efficacy Scale (GSEs), quality of life index (QL-index), etc., between the two groups at baseline, 1, 3, and 6 months will be measured.

**Discussion:**

This trial should demonstrate that the implementation of mobile-based CBT intervention will be potentially effective in lowering LDL-C levels in ASCVD patients.

**Trial registration:**

Chinese Clinical Trial Registry (ChiCTR2100046775) [registered: 2021/5/28].

## Administrative information

Note: The numbers in curly brackets in this protocol refer to SPIRIT checklist item numbers. The order of the items has been modified to group similar items. (see http://www.equator-network.org/reporting-guidelines/spirit-2727-statement-defining-standard-protocol-items-for-clinical-trials/).

**Table:** 

**Title {1}**	Effect of Cognitive Behavior Therapy (CBT) on Lowering of Blood Lipid Levels in Atherosclerotic Cardiovascular Disease (ASCVD) Patients: Study Protocol for a Multicenter, Prospective, Randomized Controlled Trial
**Trial registration {2a and 2b}.**	Chinese Clinical Trial Registry (ChiCTR2100046775) [registered: 2021/5/28].
**Protocol version {3}**	2021-July-20: Original; version 1.1.
**Funding {4}**	This trial is funded by the National Natural Science Foundation of China.
**Author details {5a}**	Xu-Lin Hong, 1028346604@zju.edu.cn; Hong-Ying Liu, hongyingliu@91jkys.com; Yi Luan, ryan1218@zju,edu,cn; Wen-Bin Zhang, 3313011@zju.edu.cn; Sir Run Run Shaw Hospital, Zhejiang University, Hangzhou, Zhejiang, China
**Name and contact information for the trial sponsor {5b}**	Jia Tang, jiatang@91jkys.com; Hangzhou Kang Sheng Health Consulting CO., LTD.
**Role of sponsor {5c}**	Hangzhou Kang Sheng Health Consulting CO., LTD provides the technology to design the MiniApp “ASCVD CBT” used in interventions. This is an investigator initiated trial. The principal investigator is also the sponsor of this trial. The principal investigator is responsible for the trial design, writing the manuscript, and leading the research team (including the nurses and physicians) to collect data.

## Introduction

### Background and rationale {6a}

Atherosclerotic cardiovascular disease (ASCVD) prevalence has been persistently increasing these years. In addition, ASCVD remains the leading cause of mortality in Chinese populations [1]. Over the past few decades, major risk factors of ASCVD have been confirmed, including the increased level of low-density lipoprotein cholesterol (LDL-C) [2]. The accumulation of LDL-C in the arterial wall can induce an inflammatory response. This leads to plaque formation and finally results in ASCVD due to plaque rupture and thrombosis. Numerous studies have consistently demonstrated that prolonged reduction of LDL-C is remarkably correlated with lowering ASCVD risk [3]. As a result, several guidelines have recommended evidence-based lipid-lowering treatments, based on lifestyle interventions, as the main components of preventing ASCVD events [3–6]. However, the issue of lower compliance to pharmacological and non-pharmacological treatments has limited the benefits of lowering lipid levels [7–9], with an estimation that 9% of ASCVD events have resulted from poor compliance [3]. In consequence, it is necessary to improve patient compliance, and furthermore, to improve the clinical outcome.

Cognitive behavior therapy (CBT) is a psychological therapy developed by A.T. Beck in the 1960s, which aims to modify out-of-control emotions, behavior, and cognition through goal-directed and systematic procedures [10]. CBT emphasizes the improvement of self-efficacy through cognitive and behavioral changes, which will benefit the patients in clinical outcomes as a result [11]. Recently, CBT has been suggested as a potential treatment to control cardiovascular disease risk factors such as weight, blood pressure, and blood glucose. Previous studies have shown that CBT can significantly reduce patients’ body weight and waist circumference [12], improve blood glucose control [13], and reduce the number of drugs required for blood pressure control [14]. Although these studies have revealed the efficacy of CBT in designated patient populations, most of the interventions are conventional methods delivered with oral communications given by healthcare providers. Study data of mobile device-based CBT used in ASCVD patients is limited. With the increasing availability and accessibility of mobile device-based healthcare, the advantage of assigned sessions, comprehensible structure, and cognitive orientation makes internet-delivered CBT a potential solution to impact a large population at a relatively low cost.

CBT-based lifestyle interventions have the potential to provide more effective patient education, leading to healthier habits at a population level. Considering these underlying factors, we assume mobile-based CBT lifestyle interventions are potentially effective in reducing LDL-C levels in ASCVD patients.

### Evidence before this study

Lifestyle modification is an evidence-based strategy associated with better clinical outcomes in ASCVD patients, accomplished with improved self-efficacy and risk factor control. Conventional lifestyle intervention is performed in an outpatient setting where healthcare providers can communicate with patients face-to-face. However, limited accessibility has been an obstacle for ASCVD patients to receive a consistent intervention. Novel delivery methods including mobile-based interventions have been developed to promote healthy lifestyles. We searched PubMed, Medline, and Embase for researches that had investigated the use of CBT in cardiovascular patients from October 2016 to October 2021. Search terms included cognitive behavioral therapy, mobile-based, cardiovascular disease, stroke, coronary syndrome, and dyslipidemia. MeSH (Medical Subject Headings) were used to include synonyms. Based on our review, we found that mobile-based CBT has been applied to improve psychological conditions in CVD patients, but few articles had investigated its improvement in objective parameters, such as lipid panels, to evaluate clinical outcomes. We identified a systemic review and meta-analysis of seventeen clinical trials (*n* = 5780) that had investigated blood pressure in patients receiving digital health interventions. The results prove that digital health interventions are effective in lowering blood pressure in hypertensive populations [15]. There is another systemic review and meta-analysis identified (56 studies; 263 effect sizes; *n* = 4060) that had investigated objective data to evaluate the improvement in immune system with psychological interventions. According to the study, psychological interventions including CBT therapy can effectively control the level of C-reactive protein in the body and slow down the progress of heart disease and Alzheimer’s disease. Among various psychological interventions, CBT is identified as superior to other psychotherapy in strengthening the immune system [16].

### Added-value of this study

CBT is widely applied to treat psychological conditions, which are mostly measured by subjective data. We identified some researches that had investigated blood pressure, C-reactive protein, etc., but there is limited information on CBT’s potential effect on lipid levels. To the best of our knowledge, this study will be the first study that evaluates CBT’s potential efficacy of reducing LDL-C levels in ASCVD patients. Moreover, this study provides a treatment that is patient-centered. Up to 50% of individuals who were involved in psychological interventions did not maximally benefit from the interventions [17]. Based on the previous studies, we consider the lack of patient-specificity is potentially limiting the efficacy. Therefore, the analysis and customization regarding specific populations are of great significance to improve the treatment selection and outcome of patients. We propose this novel treatment strategy in ASCVD patients based on the HAPA model [11]. It treats ASCVD management as a long-term process requiring patients’ self-efficacy. Customized CBT integrates mindfulness mediation, cognitive training, etc., with blood lipid management to improve self-efficacy. If the results are positive, these findings should demonstrate that the clinical improvement of mobile-based CBT intervention will be measurable as objective findings.

### Objectives {7}

Aim 1: To determine the efficacy of mobile-based CBT interventions, compared with conventional interventions, in ASCVD patients receiving standard treatments;

- Hypothesis 1a (primary study hypothesis): a significant reduction in objective parameters, including LDL-C level, will be demonstrated in participants randomly assigned to the CBT group relative to participants in the conventional interventions group.

Aim 2: To improve our capacity to match patients to treatments more efficiently by identifying self-efficacy, and quality of life.

- Hypothesis 2a: a significant improvement in self-efficacy, and quality of life, measured by scales and questionnaires, will be demonstrated in participants randomly assigned to the CBT group relative to participants in the conventional interventions group.

### Trial design {8}

This study is a multicenter, prospective, randomized, controlled, open-label parallel-group superiority trial. Patients will be randomly allocated at a 1:1 ratio to evaluate the efficacy of CBT to lower blood lipids in ASCVD patients. The study protocol follows the Standard Protocol Items: Recommendations for Interventional Trials (SPIRIT) [18].

## Methods: participants, interventions, and outcomes

### Study setting {9}

The study will be performed at Sir Run Run Shaw Hospital Affiliated to Zhejiang University, Hangzhou, Zhejiang, China, in conjunction with investigators at Ningbo Ninth Hospital, Ningbo, China, Zhejiang Xinhua Hospital, Zhejiang, China, Lin’an People’s Hospital, Zhejiang, China, and YuHang Fifth People’s Hospital, Zhejiang, China.

### Eligibility criteria {10}

#### Inclusion criteria

Patients’ inclusion criteria will be (1) age from 18 to 80 years old; (2) meet of ASCVD diagnostic criteria, including the history of acute coronary syndrome (ACS), myocardial infarction (MI), stable or unstable angina, transient ischemic attack, surgical history of coronary or peripheral vascular reconstructions, etc.; (3) currently taking statins by prescription; (4) no difficulty in using smartphones, basic Chinese reading and writing skills, basic calculation skills; and (5) acceptance of the terms and conditions of the study and signature of the informed consent form.

#### Exclusion criteria

Patients’ exclusion criteria will be (1) meet of very high-risk ASCVD diagnostic criteria (diagnostic criteria is determined according to 2020 Chinese expert consensus on lipid management of very high-risk atherosclerotic cardiovascular disease patients); (2) one or more of the following complications: heart failure, ventricular tachycardia, second to third-degree atrioventricular block, uncontrolled atrial tachycardia (including atrial flutter, atrial fibrillation, etc.), mural thrombus, cardiac aneurysm, dysfunction or rupture of papillary muscle, severe sinus bradycardia (heart rate < 50 beats/minute), sinus arrest, etc.; (3) history of cardiac arrest; (4) acute non-cardiogenic complications: such as infections, kidney failure, hyperthyroidism, etc.; (5) severe cardiovascular conditions: moderate to severe valvular stenosis, hypertrophic cardiomyopathy, other forms of ventricular outflow tract stenosis, acute myocarditis, pericarditis, active endocarditis, suspected or known aneurysm rupture, acute pulmonary embolism, or pulmonary infarction; and (6) vulnerable populations, including patients with mental disorders, critical illness, age < 18 years old, pregnancy, students or subordinate of principal investigators, employees of the research institute, etc. This trial does not exclude any other concurrent therapies or interventions.

### Who will take informed consent? {26a}

Researchers will perform an initial eligibility screening and collect written informed consent. Written informed consent will be collected prior to baseline assessment.

### Additional consent provisions for collection and use of participant data and biological specimens {26b}

If the data collection is not included in the initial informed consent process for the main clinical trial, each participant in the ancillary study must sign a consent form. This trial does not involve collecting biological specimens for storage.

### Interventions

#### Explanation for the choice of comparators {6b}

A control intervention—conventional lifestyle intervention—will be provided to help compare the effects of CBT in ASCVD patients. The control group will receive patient education delivered with oral communications given by healthcare providers. Patient education including disease-related knowledge, lifestyle guidance, complication prevention, rational drug use, and other contents will also be delivered to the control group. Patient education will be delivered only during each follow-up. Both the intervention group and the control group will receive standard pharmacological treatments.

#### Intervention description {11a}

**Fig. 1:**
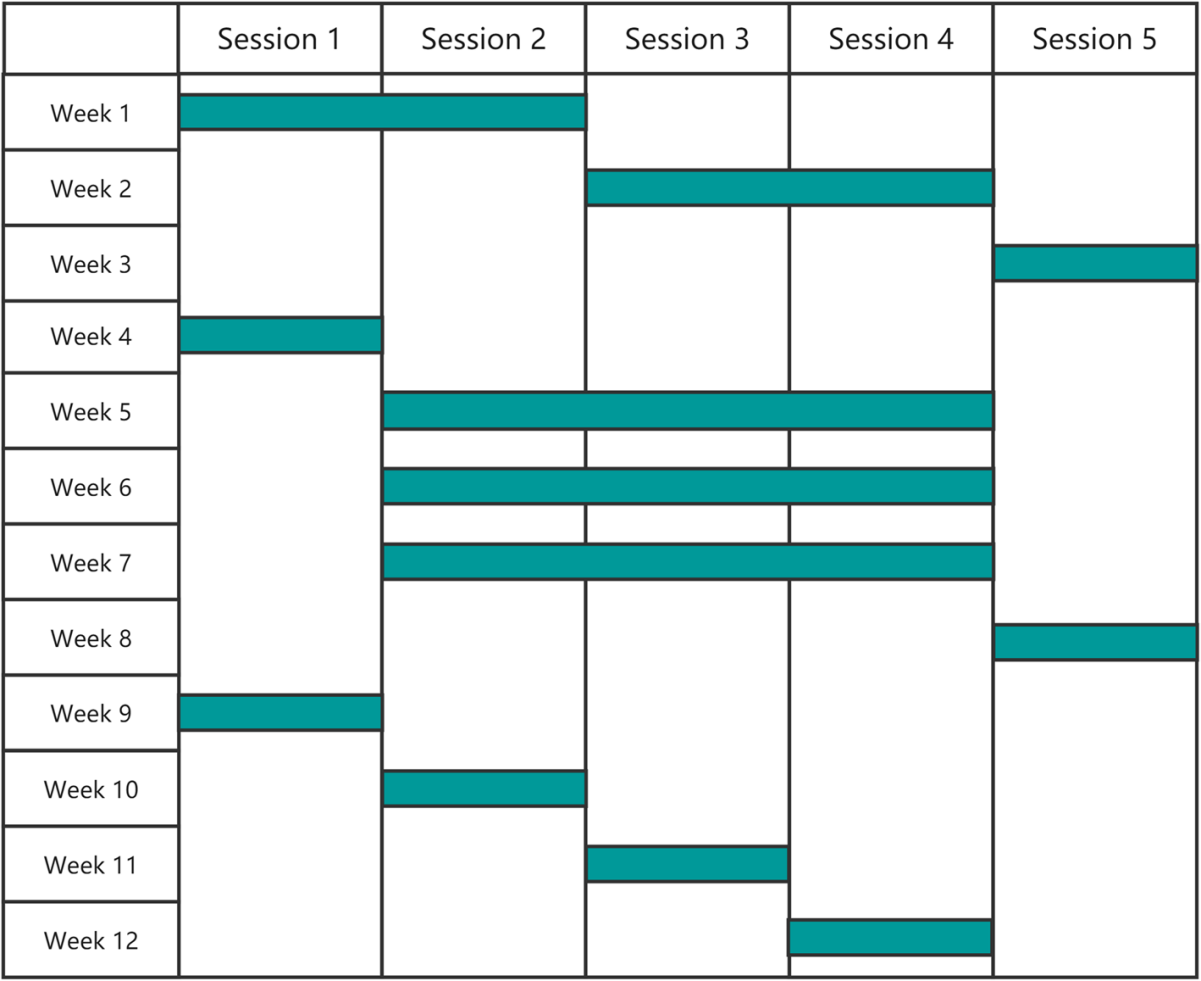
Participants’ learning schedule

CBT for ASCVD lifestyle interventions will be delivered by WeChat MiniApp: “CBT ASCVD” (developed by Hangzhou Kang Sheng Health Consulting CO., LTD). This includes 3 sections which are further divided into 6 sessions. The contents are designed and reviewed by cardiologists and psychiatrists from Sir Run Run Shaw Hospital, Zhejiang University, Hangzhou, Zhejiang, China. Participants will study according to a learning schedule which is divided into a strong intervention period (1–2 months) and a regular intervention period (4–6 months) (see Fig. 1).

#### Criteria for discontinuing or modifying allocated interventions {11b}

Discontinuation from the treatment program will be considered in the following conditions. Participants will not be considered to have dropped out of the experiment at this point, and they will be eligible to receive protocol assessments unless they withdraw their consent for assessments (as described below):

1. If the participant wishes to stop the protocol treatment;
2. If the participant is unable to continue the protocol treatment due to adverse events;
3. When the investigator believes that the risk of continuing the protocol treatment outweighs the benefit for any reason;
4. When the investigator believes that continuing the protocol treatment is not appropriate for any other reason.

#### Strategies to improve adherence to interventions {11c}

Participants in the intervention group will log in to the WeChat MiniApp to complete the intervention sessions according to the schedule. “CBT ASCVD” will track the completion of each session. To improve adherence, the participants will receive positive and constructive feedback through the “clock-in” feature in the “CBT ASCVD” app. Participants will be able to “clock in” from the dashboard to routinely track if they exercise, take medications, and eat healthy.

#### Relevant concomitant care permitted or prohibited during the trial {11d}

Both the intervention group and the control group will receive standard pharmacological treatments.

#### Provisions for post-trial care {30}

Considering the nature of mobile-based patient education, it is unlikely to cause harm to the participants. In case any risk of harm is identified, the principal investigator will intervene to minimize the potential harm. If any harm is identified, the research team will provide them with appropriate compensation if necessary.

#### Outcomes {12}

Outcomes will be collected at baseline and again at 1, 3, and 6 months during the intervention period.

### Primary outcome measure

*LDL-C level*. The primary endpoint will be the mean change in LDL-C level [time Frame: from randomization (day 0) to V5 (week 24) for a total of 6 months of treatment]. Per guideline, the LDL-C level has been validated as a primary measurement for the clinical efficacy of the treatments [3].

### Secondary outcome measures

1. The percentage of the participants with an LDL < 70 mg/dL
2. The percentage of the participants with an LDL < 70 mg/dL and an HDL > 500 mg/dL
3. The ratio of measured LDL to baseline LDL
4. Coefficient of variation of LDL
5. C-reactive protein level
6. Mean change in body mass index (BMI)
7. Mean score of General Self-Efficacy Scale (GSEs). The GSEs is a psychometric scale that assesses optimism in one’s ability to cope with a variety of life’s challenges. It consists of 10 items representing attitudes towards obstacles rated on a scale from 0 (not optimal) to 4 (very optimal). A Chinese version has been validated [19].
8. Mean score of Quality of life index (QL-index). The QL-index is a tool to evaluate quality of life [20]. It was initially established to measure lifestyle factors in cancer patients and is now commonly used in various disease states. Measurements cover: activities of daily living, principal activities, health, outlook, and support [21]. We consider QL-index as valid measurements to evaluate the quality of life in ASCVD patients.

### Participant timeline {13}

**Fig. 2:**
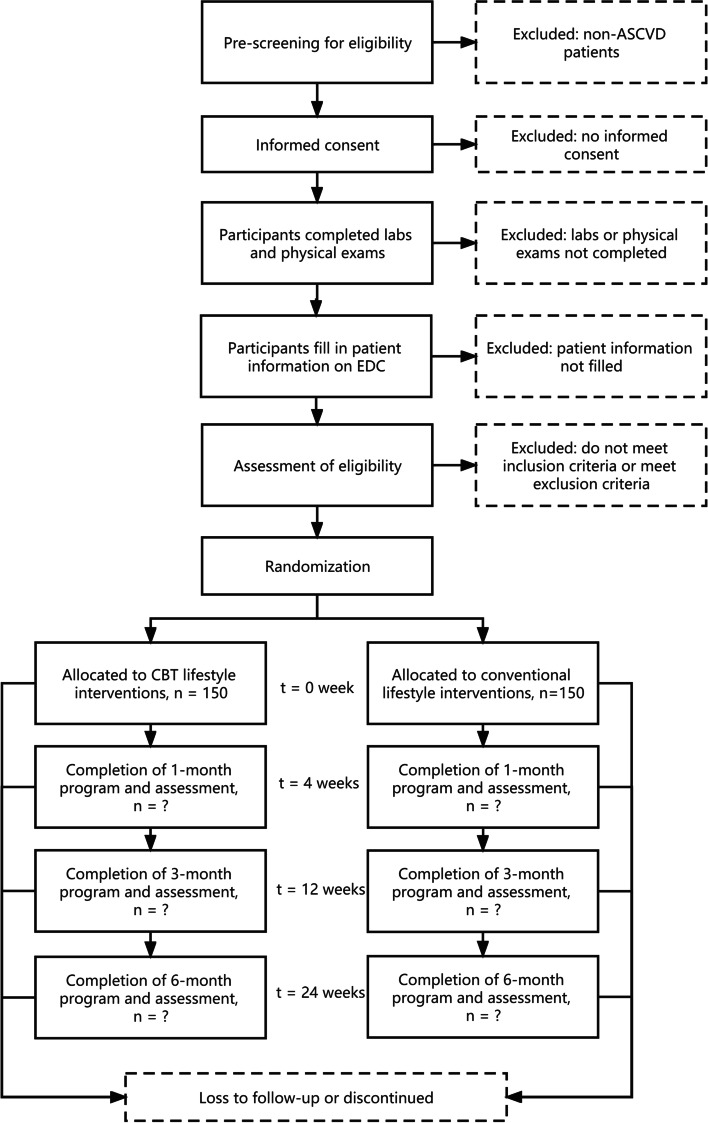
Participant timeline

The participant timeline is shown in Fig. 2.

### Sample size {14}

Considering the primary outcome variable as the reduction in the mean LDL-C from baseline to week 24, an absolute reduction of 20% in LDL-C is expected [22, 23]. One hundred twenty-two subjects per group was calculated initially to offer 80% power at a one-sided 0.05 significance level according to a 1:1 ratio of random grouping. Allowing for a maximum follow-up loss rate of 20% in both groups, 300 patients were determined as the target sample size—150 patients in each group.

### Recruitment {15}

Participant recruitment began in June 2021 and is expected to continue until 2023. We will recruit participants in 6 hospitals: Sir Run Run Shaw Hospital Affiliated to Zhejiang University, Hangzhou, Zhejiang, China, in conjunction with investigators at Ningbo Ninth Hospital, Ningbo, China, Zhejiang Xinhua Hospital, Zhejiang, China, Lin’an People’s Hospital, Zhejiang, China, and YuHang Fifth People’s Hospital, Zhejiang, China. Participants will be recruited through poster advertisements and referrals from physicians. Once recruited, researchers will teach the participants how to use the MiniApp.

### Assignment of interventions: allocation

#### Sequence generation {16a}

In this randomized controlled trial, participants will be randomized into either intervention or control arm with an allocation ratio of 1:1. The allocation will be generalized by the establishment of EDC Trial Data clinical trials centralized randomization system. Researchers will enter the system with account numbers and passwords. A randomized number generated by inputting patient information will be used to track each patient.

#### Concealment mechanism {16b}

Allocation concealment will be guaranteed through the following mechanism. Staff will log in to the EDC Trial Data cloud and enter the information for the participants. The EDC Trial Data system will randomly assign participants to either group in a 1:1 ratio. A patient profile will be generated after the necessary baseline information has been entered. Account number and password are required when assessing data.

#### Implementation {16c}

The allocation sequence will be generated by EDC centralized randomization system. XH will be in charge of enrolling the patients. The Trial Management Committee (TMC) will assign participants to interventions.

### Assignment of interventions: blinding

#### Who will be blinded {17a}

Participants and investigators will be aware of the intervention that has been assigned to them in this open-label trial. Because of the nature of the patient-reported outcome measures, not all outcome evaluations will be blinded. During the analysis, the statistician will be blinded to the allocation.

#### Procedure for unblinding if needed {17b}

The procedure for unblinding is not applicable as this is an open-label trial.

### Data collection and management

#### Plans for assessment and collection of outcomes {18a}

Data will be collected utilizing EDC in each site. Data collection takes place at four points: baseline, 1, 3, and 6 months. Collected data will be stored in the form of an electronic case report form. The clinical research coordinator (CRC) will complete the electronic case report form. The investigators will be informed of any changes made by CRC and system/edit check automatically done by the system. Authorized investigators will be permitted to access the study computers and evaluate the data throughout the study.

#### Plans to promote participant retention and complete follow-up {18b}

If any missing data is detected via the EDC system, the researcher will contact the participants by telephone.

#### Data management {19}

Study teams have a responsibility to self-monitor study processes and data. This self-monitoring can ensure a well-run trial and to identify and mitigate issues before they are identified by monitoring entities; which can result in time-consuming fixes. Internal monitoring includes monitoring for proper informed consent documentation/records, eligibility criteria, data quality, etc. Monitoring is usually conducted by interested parties involved in the research to identify issues and to improve processes. A unique identification code will be generated when analyzing data to ensure security. Errors or missing data information will be sent to data managers (DM) in Data Query Reports. DM receiving the inquiry will check the original records to determine the correction. Original paperwork and signed informed consent forms will be stored in locked file cabinets.

#### Confidentiality {27}

Personal information will be collected, shared, and maintained following ICH GCP. Before the start of the research, the principal investigator authorizes the investigators and study nurses the rights to the data set. All participants need to sign the informed consent form for the data to be collected. A randomized number is generated to track each patient. All data will be stored on a secure database (EDC). Only authorized researchers have access to the data set. Authorized researchers only have access to the data collected by themselves. Review of data collected by other researchers is not permitted.

This trial data will not be shared to enable international prospective meta-analyses.

#### Plans for collection, laboratory evaluation, and storage of biological specimens for genetic or molecular analysis in this trial/future use {33}

No genetic or molecular analysis will be conducted in this trial or for future use.

### Statistical methods

#### Statistical methods for primary and secondary outcomes {20a}

Categorical variables will be expressed in numerical values with their respective percentages and compared using the chi-squared test at baseline. Continuous variables will be expressed as mean ± SD and compared using the *t*-test. The significance of primary and secondary endpoints will be evaluated with the paired-samples *t*-test. A *p*-value < 0.05 indicates statistical significance. All data will be analyzed with the SPSS.

#### Interim analysis {21b}

There will be no interim analysis.

#### Methods for additional analysis (e.g., subgroup analysis) {20b}

No additional analysis will be conducted.

#### Methods in analysis to handle protocol non-adherence and any statistical methods to handle missing data {20c}

All patients who have received CBT for at least 4 weeks will be included in the full analysis set (FAS). Depending on the time investigated, the per-protocol (PP) analysis set will be made up of patients who complete the 6-month follow-up examinations. The FAS will be used as the basis for the primary analysis. If there is a discrepancy of greater than 10% between the FAS and PP samples, the analysis will be repeated for the PP analysis set. The safety population will include patients in the FAS. Unless otherwise noted, all analysis will be done in both the FAS and PP analysis sets.

There will be no replacement or imputation of missing values. All analysis will be based solely on observed cases. Patients who are loss to follow-up will be considered to have dropped out of therapy. Only the PP patients will be used in a sensitivity analysis to test the impact of this assumption.

#### Plans to give access to the full protocol, participant level-data, and statistical code {31c}

The results of the clinical trial will be published in the form of articles, but the specific scope and time of publication are yet to be determined. All relevant data and information will be disclosed when the article is published.

### Oversight and monitoring

#### Composition of the coordinating center and trial steering committee {5d}

*Design and conduct of the study*: preparation of protocol and amendments; preparation of IB (investigators brochure) and CRFs (case report forms); reports on studies to be published; composition of TMC’s members.

*SC (steering committee)*: final protocol agreement; patient recruitment and communication with the lead investigator; reviewing the trial’s progress and, if necessary, consenting to adjustments to the protocol and/or investigators brochure to help the study run smoothly. The steering group will include all principal investigators. A regional coordinator will be chosen from among the principal investigators at each site.

*Trial Management Committee (TMC)*: (principal investigator, research physician, administrator): study planning; organization of steering committee meetings; report of adverse events to the Chinese National Adverse Reaction Monitoring Center; responsible for the trial master file; budget management and contractual difficulties with individual centers. randomization; data verification. TMC will examine the 12 monthly feedback forms and schedule site visits; data manager: data entry and maintenance of the trial’s IT system; data verification.

*Lead investigators*: recruitment; data collection; CRF completion, as well as study patient follow-up and adherence to the study protocol and IB. A lead investigator will be assigned to each participating center and will be responsible for patient identification.

#### Composition of the data monitoring committee, its role and reporting structure {21a}

There will be no data monitoring committee as this is considered to be a low-risk intervention.

#### Adverse event reporting and harms {22}

The type and frequencies of any adverse event will be reported to the Chinese National Adverse Reaction Monitoring Center. Any trial deviations and violations will also be sent to the Chinese National Adverse Reaction Monitoring Center.

#### Frequency and plans for auditing trial conduct {23}

Audit procedures will comply with the ICH GCP (Guideline for Good Clinical Practice of the International Conference on Harmonisation) and regulatory requirements.

Project management group meeting will be conducted both weekly and monthly. Weekly meeting will be held every Friday. Monthly meeting will be conducted at the last day of every month. The project management group will audit information on the research. The ethics committee will meet every 6 months.

#### Plans for communicating important protocol amendments to relevant parties (e.g., trial participants, ethical committees) {25}

The steering committee will determine any revisions of the protocol. The amended protocol will also be submitted to the Medical Ethics Committee of the Sir Run Run Shaw Hospital of Zhejiang University and reported to the participants as necessary.

#### Dissemination plans {31a}

The findings of the study will be presented to the public at academic conferences. The steering group will decide on authorship. The order of the authors will be determined by the contributions of each member.

## Discussion

This article presents a detailed description of a multicenter RCT, designed to evaluate the effectiveness of CBT to reduce LDL-C levels in ASCVD patients. The treatment for ASCVD generally involves long-term lifestyle management at home. To accomplish the primary goal of controlling blood lipids, we consider the mobile-based CBT intervention as an available and accessible method to promote healthy habits, and as a result, this will potentially lower LDL-C in ASCVD patients. We believe that mobile-based CBT lifestyle intervention will be potentially effective in lowering LDL-C levels in ASCVD patients.

This study has some critical implications. The novel mobile-based intervention is expected to reduce LDL-C level at a population level due to the high accessibility and availability of technologies. Moreover, this CBT treatment can potentially fill the existing gaps of limited professional lifestyle interventions in ASCVD patients.

There are some limitations of this study. First, it is impossible to blind participants and staff (excluding statistician) in psychotherapy studies due to the nature of the research. As a result, therapy expectancy effects may be a source of bias. Second, mobile-based delivery method may anticipate unexpected challenges including technology issues. We will keep in track any technology difficulties for the participants and any technology issues that happen during the study, to advise mobile-based delivery improvement in the future.

### Trial status

Protocol version: original; version 1.1 20210720

Recruitment start date: August 1, 2021

Anticipated completion of recruitment: December 31, 2021

Actual completion of recruitment: January 2022

## Abbreviations

ACS: Acute coronary syndrome
ASCVD: Atherosclerotic cardiovascular disease
BMI: Body mass index
CBT: Cognitive behavioral therapy
CRC: Clinical research coordinator
CRFs: Case report forms
DM: Data manager
FAS: Full analysis set
GEGS: General Self-Efficacy Scale (GSEs)
HIPAA: Health Insurance Portability and Accountability Act
IB: Investigators brochure
LDL-C: Low-density lipoprotein cholesterol
ICH GCP: Guideline for Good Clinical Practice of the International Conference on Harmonisation
MeSH: Medical subject headings
MI: Myocardial infarction
PP: Per-protocol
QL-index: Quality of life index
SC: Steering committee
SPIRIT: Standard Protocol Items Recommendations for Interventional Trials
TMC: Trial management committee
